# *rac*-3-[(Anilino)(naphthalen-2-yl)­methyl]thian-4-one

**DOI:** 10.1107/S1600536812005983

**Published:** 2012-02-17

**Authors:** Klaus Harms, M. Saeed Abaee, Mohammad M. Mojtahedi, A. Wahid Mesbah

**Affiliations:** a Fachbereich Chemie, Philipps Universität Marburg, Hans Meerwein Strasse, Marburg, D-35032, Germany; b Department of Organic Chemistry, Chemistry and Chemical Engineering Research Center of Iran, PO Box 14335-186, Tehran, Iran

## Abstract

In the title compound, C_22_H_21_NOS, the thio­pyran­one ring adopts a chair-like conformation with the substituent in the axial position. The relative configuration of the racemic compound is 3*R*,7*S* according to the numbering scheme used in this publication. In the crystal packing, centrosymmetric dimers are built up *via* N—H⋯O hydrogen bonds, with graph set *R*_2_^2^(8).

## Related literature

For the preparation and spectroscopic characterization of the title compound and a series of related compounds, see: Abaee *et al.* (2012 ▶). For the crystal structure of *rac*-3-[(3-chloro­anilino)(4-chlorophenyl)methyl]thian-4-one, see: Harms *et al.* (2012 ▶). For the crystal structures of related compounds, see: Guo *et al.* (2007 ▶); Fun *et al.* (2009 ▶). For patterns in hydrogen bonding, see: Bernstein *et al.* (1995 ▶).
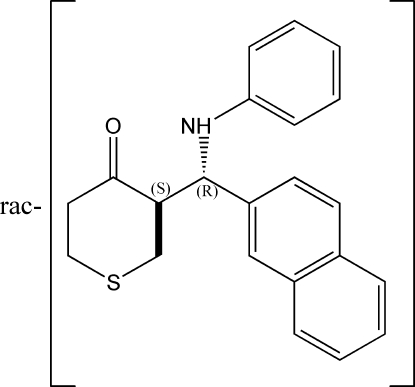


## Experimental

### 

#### Crystal data

C_22_H_21_NOS
*M* *_r_* = 347.46
Monoclinic, 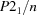

*a* = 10.8049 (10) Å
*b* = 10.5497 (15) Å
*c* = 16.4936 (16) Å
β = 97.141 (8)°
*V* = 1865.5 (4) Å^3^
*Z* = 4
Mo *K*α radiation
μ = 0.18 mm^−1^
*T* = 193 K
0.45 × 0.45 × 0.36 mm

#### Data collection

Stoe IPDS I diffractometer
Absorption correction: integration [*X-AREA* and *X-RED32* (Stoe & Cie, 2006 ▶)] *T*_min_ = 0.942, *T*_max_ = 0.960
13547 measured reflections
3244 independent reflections
1939 reflections with *I* > 2σ(*I*)
*R*_int_ = 0.044

#### Refinement

*R*[*F* ^2^ > 2σ(*F* ^2^)] = 0.033
*wR*(*F* ^2^) = 0.074
*S* = 0.79
3244 reflections
230 parameters
H atoms treated by a mixture of independent and constrained refinement
Δρ_max_ = 0.23 e Å^−3^
Δρ_min_ = −0.37 e Å^−3^

### 

Data collection: *EXPOSE* (Stoe & Cie, 1994 ▶); cell refinement: *CELL* (Stoe & Cie, 1994 ▶); data reduction: *X-RED32* (Stoe & Cie, 2006 ▶); program(s) used to solve structure: *SIR92* (Altomare *et al.*, 1994 ▶); program(s) used to refine structure: *SHELXL97* (Sheldrick, 2008 ▶); molecular graphics: *DIAMOND* (Brandenburg, 2007 ▶); software used to prepare material for publication: *publCIF* (Westrip, 2010 ▶), *PLATON* (Spek, 2009 ▶), and *WinGX* (Farrugia, 1999 ▶).

## Supplementary Material

Crystal structure: contains datablock(s) I, global. DOI: 10.1107/S1600536812005983/fj2509sup1.cif

Structure factors: contains datablock(s) I. DOI: 10.1107/S1600536812005983/fj2509Isup2.hkl

Supplementary material file. DOI: 10.1107/S1600536812005983/fj2509Isup3.cml

Additional supplementary materials:  crystallographic information; 3D view; checkCIF report

## Figures and Tables

**Table 1:** Hydrogen-bond geometry (Å, °)

*D*—H⋯*A*	*D*—H	H⋯*A*	*D*⋯*A*	*D*—H⋯*A*
N8—H8⋯O1^i^	0.926 (15)	2.121 (16)	3.0450 (18)	175.4 (13)

Symmetry code: (i) 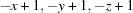.

**Fig. 1.**
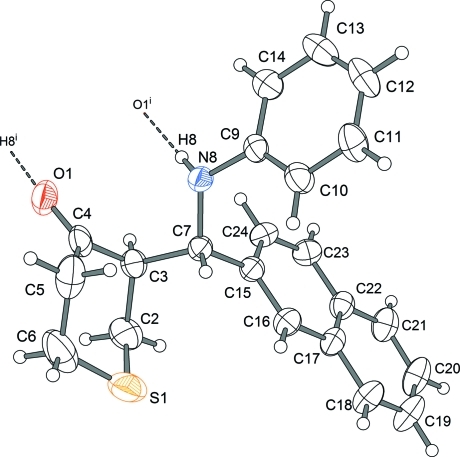
Molecular structure of the title compound with the atom numbering scheme. Displacement ellipsoids are drawn at 50% probability level. H atoms are presented as small spheres of arbitrary radius. Dotted lines indicate hydrogen bonds to the neighbouring molecule generated by crystallographic inversion symmetry. For symmetry code (i), see Table 1.

## supplementary crystallographic information

### Experimental

The title compound is an example of a series of products of an *anti*-selective three-component Mannich reaction in the thiopyran-4-one system; see Abaee *et al.* (2012) for details. Colourless crystals suitable for crystal structure determination were grown from ethyl acetate.

### Refinement

Data have been merged using the program *X-RED32* (Stoe & Cie, 2006). Three beamstop affected reflections (1 1 0, -1 1 1, 0 1 1) have been excluded from the data during the refinement. All C bonded H atoms were placed in geometrical positions and constrained to ride on their parent atoms with C—H distances in the range 0.95–1.00 Å. The *U*_iso_ values were constrained to be 1.2*U*_eq_ of the parent C atom. The position of the N bonded H atom has been refined freely with an isotropic displacement factor. The N—H bond length is 0.926 (15) Å.

### Crystal data

**Table:**

C_22_H_21_NOS	*F*(000) = 736
*M**_r_* = 347.46	*D*_x_ = 1.237 Mg m^−^^3^
Monoclinic, *P*2_1_/*n*	Mo *K*α radiation, λ = 0.71073 Å
Hall symbol: -P 2yn	Cell parameters from 7999 reflections
*a* = 10.8049 (10) Å	θ = 2.3–26.0°
*b* = 10.5497 (15) Å	µ = 0.18 mm^−^^1^
*c* = 16.4936 (16) Å	*T* = 193 K
β = 97.141 (8)°	Nugget, colourless
*V* = 1865.5 (4) Å^3^	0.45 × 0.45 × 0.36 mm
*Z* = 4	

### Data collection

**Table:**

Stoe IPDS I diffractometer	3244 independent reflections
Radiation source: fine-focus sealed tube	1939 reflections with *I* > 2σ(*I*)
Graphite monochromator	*R*_int_ = 0.044
Detector resolution: 6.67 pixels mm^-1^	θ_max_ = 25.4°, θ_min_ = 2.1°
rotation method scans	*h* = −13→12
Absorption correction: integration [*X-AREA* and *X-RED32* (Stoe & Cie, 2006)]	*k* = −12→12
*T*_min_ = 0.942, *T*_max_ = 0.960	*l* = −19→19
13547 measured reflections	

### Refinement

**Table:**

Refinement on *F*^2^	Primary atom site location: structure-invariant direct methods
Least-squares matrix: full	Secondary atom site location: difference Fourier map
*R*[*F*^2^ > 2σ(*F*^2^)] = 0.033	Hydrogen site location: inferred from neighbouring sites
*wR*(*F*^2^) = 0.074	H atoms treated by a mixture of independent and constrained refinement
*S* = 0.79	*w* = 1/[σ^2^(*F*_o_^2^) + (0.0399*P*)^2^] where *P* = (*F*_o_^2^ + 2*F*_c_^2^)/3
3244 reflections	(Δ/σ)_max_ < 0.001
230 parameters	Δρ_max_ = 0.23 e Å^−^^3^
0 restraints	Δρ_min_ = −0.37 e Å^−^^3^

### Special details

**Table:**

Geometry. All e.s.d.'s (except the e.s.d. in the dihedral angle between two l.s. planes) are estimated using the full covariance matrix. The cell e.s.d.'s are taken into account individually in the estimation of e.s.d.'s in distances, angles and torsion angles; correlations between e.s.d.'s in cell parameters are only used when they are defined by crystal symmetry. An approximate (isotropic) treatment of cell e.s.d.'s is used for estimating e.s.d.'s involving l.s. planes.
Refinement. Refinement of *F*^2^ against ALL reflections. The weighted *R*-factor *wR* and goodness of fit *S* are based on *F*^2^, conventional *R*-factors *R* are based on *F*, with *F* set to zero for negative *F*^2^. The threshold expression of *F*^2^ > σ(*F*^2^) is used only for calculating *R*-factors(gt) *etc*. and is not relevant to the choice of reflections for refinement. *R*-factors based on *F*^2^ are statistically about twice as large as those based on *F*, and *R*-factors based on ALL data will be even larger.

### Fractional atomic coordinates and isotropic or equivalent isotropic displacement parameters (Å^2^)

**Table:**

	*x*	*y*	*z*	*U*_iso_*/*U*_eq_	
S1	0.06564 (5)	0.68462 (6)	0.53056 (4)	0.0733 (2)	
O1	0.38884 (11)	0.55992 (11)	0.42256 (7)	0.0481 (3)	
N8	0.48932 (11)	0.69693 (12)	0.59618 (8)	0.0318 (3)	
H8	0.5282 (14)	0.6206 (15)	0.5880 (10)	0.033 (4)*	
C2	0.16654 (16)	0.55096 (17)	0.55576 (14)	0.0568 (6)	
H2A	0.1584	0.5237	0.6123	0.068*	
H2B	0.1386	0.4798	0.5188	0.068*	
C3	0.30417 (14)	0.57832 (15)	0.54910 (11)	0.0359 (4)	
H3	0.3517	0.4977	0.5611	0.043*	
C4	0.32089 (15)	0.61845 (15)	0.46296 (11)	0.0381 (4)	
C5	0.24585 (19)	0.72936 (18)	0.42754 (13)	0.0579 (6)	
H5A	0.2618	0.7424	0.3703	0.070*	
H5B	0.2728	0.8067	0.4588	0.070*	
C6	0.1050 (2)	0.7089 (2)	0.42969 (16)	0.0808 (8)	
H6A	0.0776	0.6344	0.3956	0.097*	
H6B	0.0591	0.7838	0.4055	0.097*	
C7	0.36003 (13)	0.67883 (14)	0.61120 (10)	0.0301 (4)	
H7	0.3141	0.7603	0.5991	0.036*	
C9	0.56671 (14)	0.78247 (14)	0.64329 (10)	0.0306 (4)	
C10	0.52229 (16)	0.89433 (15)	0.67309 (11)	0.0414 (4)	
H10	0.4354	0.9118	0.6656	0.050*	
C11	0.60450 (18)	0.98055 (17)	0.71379 (12)	0.0519 (5)	
H11	0.5731	1.0574	0.7333	0.062*	
C12	0.73045 (18)	0.95712 (18)	0.72650 (13)	0.0542 (5)	
H12	0.7860	1.0172	0.7541	0.065*	
C13	0.77470 (17)	0.84535 (18)	0.69860 (12)	0.0494 (5)	
H13	0.8615	0.8275	0.7078	0.059*	
C14	0.69458 (15)	0.75867 (16)	0.65734 (11)	0.0396 (4)	
H14	0.7268	0.6819	0.6383	0.047*	
C15	0.34684 (14)	0.64065 (13)	0.69838 (10)	0.0309 (4)	
C16	0.25886 (15)	0.69614 (14)	0.74005 (11)	0.0362 (4)	
H16	0.2072	0.7609	0.7141	0.043*	
C17	0.24295 (15)	0.65944 (15)	0.82070 (11)	0.0358 (4)	
C18	0.14989 (17)	0.71312 (17)	0.86377 (12)	0.0484 (5)	
H18	0.0965	0.7772	0.8386	0.058*	
C19	0.1364 (2)	0.67372 (18)	0.94068 (12)	0.0561 (6)	
H19	0.0730	0.7099	0.9685	0.067*	
C20	0.21464 (19)	0.58064 (17)	0.97926 (12)	0.0551 (6)	
H20	0.2039	0.5538	1.0329	0.066*	
C21	0.30610 (18)	0.52812 (16)	0.94044 (11)	0.0457 (5)	
H21	0.3597	0.4660	0.9677	0.055*	
C22	0.32231 (15)	0.56508 (14)	0.85973 (11)	0.0353 (4)	
C23	0.41391 (15)	0.51076 (15)	0.81611 (11)	0.0385 (4)	
H23	0.4686	0.4481	0.8417	0.046*	
C24	0.42500 (14)	0.54655 (14)	0.73845 (11)	0.0354 (4)	
H24	0.4866	0.5076	0.7104	0.042*	

### Atomic displacement parameters (Å^2^)

**Table:**

	*U*^11^	*U*^22^	*U*^33^	*U*^12^	*U*^13^	*U*^23^
S1	0.0349 (2)	0.0790 (4)	0.1041 (6)	−0.0052 (3)	0.0016 (3)	−0.0435 (4)
O1	0.0546 (7)	0.0537 (7)	0.0350 (8)	0.0127 (6)	0.0011 (7)	−0.0116 (6)
N8	0.0310 (7)	0.0330 (7)	0.0325 (9)	−0.0052 (6)	0.0084 (6)	−0.0056 (6)
C2	0.0460 (11)	0.0515 (11)	0.0727 (16)	−0.0199 (9)	0.0067 (11)	−0.0201 (10)
C3	0.0378 (9)	0.0311 (8)	0.0383 (12)	−0.0046 (7)	0.0032 (8)	−0.0072 (7)
C4	0.0410 (9)	0.0359 (9)	0.0348 (12)	−0.0004 (8)	−0.0054 (9)	−0.0129 (8)
C5	0.0824 (14)	0.0510 (11)	0.0378 (13)	0.0241 (10)	−0.0030 (11)	−0.0043 (9)
C6	0.0664 (14)	0.0796 (16)	0.085 (2)	0.0312 (12)	−0.0363 (13)	−0.0339 (14)
C7	0.0305 (8)	0.0311 (8)	0.0293 (10)	−0.0030 (7)	0.0062 (7)	−0.0028 (7)
C9	0.0353 (8)	0.0343 (8)	0.0222 (10)	−0.0076 (7)	0.0031 (8)	0.0042 (7)
C10	0.0392 (9)	0.0390 (9)	0.0449 (13)	−0.0053 (8)	0.0005 (9)	−0.0058 (8)
C11	0.0577 (12)	0.0432 (10)	0.0518 (15)	−0.0085 (9)	−0.0048 (11)	−0.0117 (9)
C12	0.0549 (12)	0.0532 (12)	0.0490 (15)	−0.0191 (10)	−0.0154 (10)	−0.0005 (10)
C13	0.0380 (9)	0.0556 (12)	0.0508 (14)	−0.0110 (9)	−0.0100 (9)	0.0116 (10)
C14	0.0399 (9)	0.0402 (9)	0.0379 (12)	−0.0031 (8)	0.0022 (9)	0.0073 (8)
C15	0.0303 (8)	0.0294 (8)	0.0337 (11)	−0.0061 (7)	0.0067 (8)	−0.0040 (7)
C16	0.0391 (8)	0.0338 (9)	0.0364 (12)	0.0022 (7)	0.0077 (8)	−0.0008 (8)
C17	0.0424 (9)	0.0331 (9)	0.0336 (11)	−0.0021 (7)	0.0118 (9)	−0.0048 (7)
C18	0.0591 (11)	0.0453 (10)	0.0440 (13)	0.0128 (9)	0.0188 (10)	−0.0016 (9)
C19	0.0763 (14)	0.0537 (11)	0.0440 (14)	0.0113 (11)	0.0299 (12)	−0.0060 (10)
C20	0.0856 (15)	0.0481 (11)	0.0354 (12)	0.0026 (11)	0.0228 (12)	−0.0021 (9)
C21	0.0627 (12)	0.0381 (9)	0.0371 (12)	0.0003 (9)	0.0097 (10)	−0.0001 (8)
C22	0.0419 (9)	0.0314 (8)	0.0336 (11)	−0.0044 (7)	0.0082 (8)	−0.0027 (7)
C23	0.0382 (9)	0.0347 (9)	0.0432 (13)	0.0030 (7)	0.0078 (9)	0.0023 (8)
C24	0.0325 (8)	0.0356 (9)	0.0398 (12)	0.0012 (7)	0.0114 (8)	0.0000 (8)

### Geometric parameters (Å, º)

**Table:**

S1—C6	1.786 (3)	C11—H11	0.9500
S1—C2	1.799 (2)	C12—C13	1.373 (3)
O1—C4	1.2190 (18)	C12—H12	0.9500
N8—C9	1.398 (2)	C13—C14	1.379 (2)
N8—C7	1.4614 (18)	C13—H13	0.9500
N8—H8	0.926 (15)	C14—H14	0.9500
C2—C3	1.532 (2)	C15—C16	1.372 (2)
C2—H2A	0.9900	C15—C24	1.413 (2)
C2—H2B	0.9900	C16—C17	1.416 (2)
C3—C4	1.515 (2)	C16—H16	0.9500
C3—C7	1.544 (2)	C17—C22	1.415 (2)
C3—H3	1.0000	C17—C18	1.419 (2)
C4—C5	1.500 (2)	C18—C19	1.360 (3)
C5—C6	1.542 (3)	C18—H18	0.9500
C5—H5A	0.9900	C19—C20	1.397 (3)
C5—H5B	0.9900	C19—H19	0.9500
C6—H6A	0.9900	C20—C21	1.361 (2)
C6—H6B	0.9900	C20—H20	0.9500
C7—C15	1.517 (2)	C21—C22	1.419 (2)
C7—H7	1.0000	C21—H21	0.9500
C9—C10	1.387 (2)	C22—C23	1.415 (2)
C9—C14	1.395 (2)	C23—C24	1.355 (2)
C10—C11	1.385 (2)	C23—H23	0.9500
C10—H10	0.9500	C24—H24	0.9500
C11—C12	1.373 (3)		
			
C6—S1—C2	96.89 (10)	C12—C11—C10	121.29 (18)
C9—N8—C7	120.55 (12)	C12—C11—H11	119.4
C9—N8—H8	113.1 (10)	C10—C11—H11	119.4
C7—N8—H8	111.8 (9)	C13—C12—C11	118.89 (17)
C3—C2—S1	113.67 (13)	C13—C12—H12	120.6
C3—C2—H2A	108.8	C11—C12—H12	120.6
S1—C2—H2A	108.8	C12—C13—C14	120.76 (17)
C3—C2—H2B	108.8	C12—C13—H13	119.6
S1—C2—H2B	108.8	C14—C13—H13	119.6
H2A—C2—H2B	107.7	C13—C14—C9	120.70 (16)
C4—C3—C2	110.49 (16)	C13—C14—H14	119.7
C4—C3—C7	110.36 (12)	C9—C14—H14	119.7
C2—C3—C7	112.62 (13)	C16—C15—C24	118.46 (15)
C4—C3—H3	107.7	C16—C15—C7	120.95 (14)
C2—C3—H3	107.7	C24—C15—C7	120.59 (13)
C7—C3—H3	107.7	C15—C16—C17	121.78 (15)
O1—C4—C5	121.02 (17)	C15—C16—H16	119.1
O1—C4—C3	121.42 (15)	C17—C16—H16	119.1
C5—C4—C3	117.49 (15)	C22—C17—C16	118.82 (14)
C4—C5—C6	111.66 (17)	C22—C17—C18	118.67 (16)
C4—C5—H5A	109.3	C16—C17—C18	122.51 (16)
C6—C5—H5A	109.3	C19—C18—C17	120.67 (17)
C4—C5—H5B	109.3	C19—C18—H18	119.7
C6—C5—H5B	109.3	C17—C18—H18	119.7
H5A—C5—H5B	107.9	C18—C19—C20	120.71 (17)
C5—C6—S1	113.05 (16)	C18—C19—H19	119.6
C5—C6—H6A	109.0	C20—C19—H19	119.6
S1—C6—H6A	109.0	C21—C20—C19	120.40 (18)
C5—C6—H6B	109.0	C21—C20—H20	119.8
S1—C6—H6B	109.0	C19—C20—H20	119.8
H6A—C6—H6B	107.8	C20—C21—C22	120.76 (18)
N8—C7—C15	113.57 (13)	C20—C21—H21	119.6
N8—C7—C3	106.28 (12)	C22—C21—H21	119.6
C15—C7—C3	111.80 (12)	C23—C22—C17	118.42 (15)
N8—C7—H7	108.3	C23—C22—C21	122.78 (16)
C15—C7—H7	108.3	C17—C22—C21	118.79 (15)
C3—C7—H7	108.3	C24—C23—C22	121.16 (15)
C10—C9—C14	118.27 (15)	C24—C23—H23	119.4
C10—C9—N8	122.46 (14)	C22—C23—H23	119.4
C14—C9—N8	119.17 (14)	C23—C24—C15	121.34 (14)
C11—C10—C9	120.08 (16)	C23—C24—H24	119.3
C11—C10—H10	120.0	C15—C24—H24	119.3
C9—C10—H10	120.0		
			
C6—S1—C2—C3	57.57 (16)	N8—C9—C14—C13	175.40 (15)
S1—C2—C3—C4	−59.40 (17)	N8—C7—C15—C16	135.46 (15)
S1—C2—C3—C7	64.52 (19)	C3—C7—C15—C16	−104.28 (16)
C2—C3—C4—O1	−121.46 (17)	N8—C7—C15—C24	−44.72 (19)
C7—C3—C4—O1	113.33 (16)	C3—C7—C15—C24	75.54 (18)
C2—C3—C4—C5	55.62 (19)	C24—C15—C16—C17	−1.5 (2)
C7—C3—C4—C5	−69.58 (19)	C7—C15—C16—C17	178.33 (14)
O1—C4—C5—C6	121.38 (19)	C15—C16—C17—C22	1.6 (2)
C3—C4—C5—C6	−55.7 (2)	C15—C16—C17—C18	−178.03 (16)
C4—C5—C6—S1	59.1 (2)	C22—C17—C18—C19	−0.7 (3)
C2—S1—C6—C5	−56.56 (16)	C16—C17—C18—C19	178.91 (18)
C9—N8—C7—C15	−56.27 (19)	C17—C18—C19—C20	0.7 (3)
C9—N8—C7—C3	−179.60 (14)	C18—C19—C20—C21	0.2 (3)
C4—C3—C7—N8	−54.62 (17)	C19—C20—C21—C22	−1.1 (3)
C2—C3—C7—N8	−178.61 (15)	C16—C17—C22—C23	−0.4 (2)
C4—C3—C7—C15	−179.05 (13)	C18—C17—C22—C23	179.22 (15)
C2—C3—C7—C15	56.96 (19)	C16—C17—C22—C21	−179.83 (15)
C7—N8—C9—C10	−34.1 (2)	C18—C17—C22—C21	−0.2 (2)
C7—N8—C9—C14	149.59 (15)	C20—C21—C22—C23	−178.28 (17)
C14—C9—C10—C11	1.6 (2)	C20—C21—C22—C17	1.1 (3)
N8—C9—C10—C11	−174.77 (16)	C17—C22—C23—C24	−0.8 (2)
C9—C10—C11—C12	−0.8 (3)	C21—C22—C23—C24	178.61 (17)
C10—C11—C12—C13	−0.5 (3)	C22—C23—C24—C15	0.9 (2)
C11—C12—C13—C14	1.0 (3)	C16—C15—C24—C23	0.3 (2)
C12—C13—C14—C9	−0.2 (3)	C7—C15—C24—C23	−179.57 (15)
C10—C9—C14—C13	−1.1 (2)		

### Hydrogen-bond geometry (Å, º)

**Table:**

*D*—H···*A*	*D*—H	H···*A*	*D*···*A*	*D*—H···*A*
N8—H8···O1^i^	0.926 (15)	2.121 (16)	3.0450 (18)	175.4 (13)

Symmetry code: (i) −*x*+1, −*y*+1, −*z*+1.
